# Detection of genomic rearrangements from targeted resequencing data in Parkinson's disease patients

**DOI:** 10.1002/mds.26845

**Published:** 2016-11-07

**Authors:** Nino Spataro, Ana Roca‐Umbert, Laura Cervera‐Carles, Mònica Vallès, Roger Anglada, Javier Pagonabarraga, Berta Pascual‐Sedano, Antònia Campolongo, Jaime Kulisevsky, Ferran Casals, Jordi Clarimón, Elena Bosch

**Affiliations:** ^1^ Institute of Evolutionary Biology (CSIC‐UPF), Department of Experimental and Health SciencesUniversitat Pompeu FabraBarcelonaSpain; ^2^ Department of Neurology, Institut d'Investigacions Biomèdiques Sant Pau‐Hospital de la Santa Creu i Sant PauUniversitat Autònoma de BarcelonaBarcelonaSpain; ^3^ Center for Networking Biomedical Research in Neurodegenerative Diseases (CIBERNED)MadridSpain; ^4^ Genomics Core FacilityUniversitat Pompeu Fabra, Barcelona Biomedical Research Park (PRBB)BarcelonaSpain; ^5^ Health Sciences DepartmentUniversitat Oberta de CatalunyaCataloniaSpain

**Keywords:** Parkinson's disease, next generation sequencing, structural variants, XHMM software

## Abstract

**Background:**

The analysis of coverage depth in next‐generation sequencing data allows the detection of gene dose alterations. We explore the frequency of such structural events in a Spanish cohort of sporadic PD cases.

**Methods:**

Gene dose alterations were detected with the eXome‐Hidden Markov Model (XHMM) software from depth of coverage in resequencing data available for 38 Mendelian and other risk PD loci in 394 individuals (249 cases and 145 controls) and subsequently validated by quantitative PCR.

**Results:**

We identified 10 PD patients with exon dosage alterations in *PARK2, GBA‐GBAP1*, and *DJ1*. Additional functional variants, including 2 novel nonsense mutations (p.Arg1552Ter in *LRRK2* and p.Trp90Ter in *PINK1*), were confirmed by Sanger sequencing. This combined approach disclosed the genetic cause of 12 PD cases.

**Conclusions:**

Gene dose alterations related to PD can be correctly identified from targeting resequencing data. This approach substantially improves the detection rate of cases with causal genetic alterations. © 2016 The Authors. Movement Disorders published by Wiley Periodicals, Inc. on behalf of International Parkinson and Movement Disorder Society.

Next‐generation sequencing (NGS) technologies such as whole‐genome, whole‐exome, and custom targeting sequencing strategies are increasingly being used to understand the genetic factors underlying both common and rare neurological disorders.^1^, ^2^ Notably, the recent advent of the NGS technologies has been accompanied by the implementation of new bioinformatic tools to detect copy number variants (CNVs) from the depth of read mapping, even after applying specific targeted enrichment.^3^, ^4^, ^5^, ^6^, ^7^ Most of these new tools are based on the assumption that differences in the depth of coverage among specific genomic regions and across multiple samples can be used as an indicator of the relative number of copies, resulting in a semiquantitative estimation of CNVs. However, as far as we know, this approach has never been attempted in PD.

The main aim of this study was to explore whether CNVs help to explain some sporadic PD patients in which no causative point mutations are found. In particular, we used our own resequencing data of 38 PD‐associated genes in 249 PD cases and 145 unrelated controls of European ancestry^8^ to study the presence of structural variants predicted by eXome‐Hidden Markov Model (XHMM) software (https://atgu.mgh.harvard.edu/xhmm/), which was specifically developed to recover information on copy number variation from normalized read depth data obtained from targeted sequencing experiments.^3^ Subsequently, all the predicted CNVs were validated through quantitative polymerase chain reaction (PCR). Moreover, detailed frequencies for all potentially functional exon dose alterations detected here and for previously described pathogenic single nucleotide polymorphisms (SNPs) and indels in the same resequencing dataset are provided to understand their relative relevance in PD.

## Methods

### Participants and Targeted Resequencing Data

Targeted resequencing data from 394 individuals including 249 idiopathic PD cases and 145 unrelated controls of European origin were compiled from Spataro et al. (2015).^8^ Details on eligibility criteria, clinical and demographic features of PD patients and targeting resequencing design are available in Appendix 1 (Supplementary Data).

### Coverage and Detection of CNVs

The mean coverage per target and sample was 49.39X, and 91% of the target bases were covered at ≥15X depth.^8^ The detection of CNVs was performed with the XHMM software, which uses principal component analysis normalization and a hidden Markov model to detect and genotype CNVs from normalized read‐depth data from targeted sequencing experiments.^3^ Phred‐scaled quality scores for the CNV events in the inferred intervals ranged from 30 to 99, with a mean of 80 (see Table S1). Gene dose alterations in Mendelian PD genes were then subsequently validated by quantitative PCR (see details in Appendix 2 in the Supplementary Data).

### PCR and Sanger Sequencing Validation Analysis

Frameshift deletions and stop gain mutations within the *PARK2*, *LRRK2* and *PINK1* genes plus genomic rearrangements around the *GBA‐GBAP1* region were confirmed by PCR and Sanger sequencing analyses (see details in Appendix 3 in the Supplementary Data).

### Collapsing Tests

The potential enrichment of exon dosage alterations was tested in the whole set of cases and controls using the full list of CNVs reported in Table S1. CNV enrichment was performed with the VariantTools package (http://varianttools.sourceforge.net/), which includes up to 12 different collapsing tests.^9^

## Results

**Figure 1:**
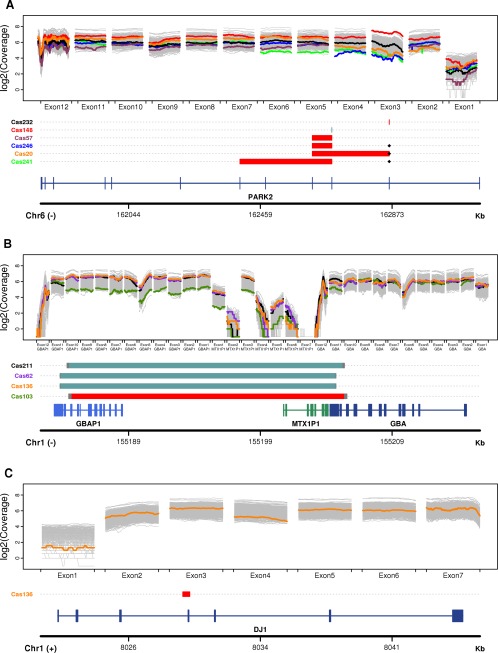
Detection of copy number variation: (**A**) *PARK2* gene, (**B**) *GBA‐GBAP1* region, and (**C**) *DJ1* gene. In the upper panel, sequencing depth of coverage for those samples inferred to carry copy number variants by the eXome‐Hidden Markov Model (XHMM) software (https://atgu.mgh.harvard.edu/xhmm/) is indicated in colors along each exon; the color matches that of the individual label on the *Y* axis of the bottom panel. In all other samples, the gray background represents sequencing depth of coverage. In the bottom panel, a schematic representation of the corresponding validated copy number variants is presented for each gene. Red bars, deletion; light blue bars, duplication; black diamonds, frameshift indels. The bottom track represents a schematic representation of the gene structure.

Our study of structural genetic alterations across the 38 PD‐associated genes previously sequenced through NGS^8^ disclosed a total of 11 structural variants in the *PARK2*, *GBA,* and *DJ1* genes, affecting 10 of 249 PD cases. All of these genomic alterations were predicted by the XHMM sofware^3^ (see details in Table S1) and were subsequently validated by quantitative PCR (Figure 1 and Figure S1). CNVs were also detected in 2 of 145 controls in a heterozygous state for the recessive gene *PARK2* and the *GBA‐GBAP1* region. The clinical features of the PD samples in which structural variants were found are provided in Appendix 1 (Supplementary Data). Additional functional sequence alterations detected in the same dataset are listed in Table 1 (see Table S2 for complete genotypes).

**Table 1:** Summary of pathogenic alterations found in Mendelian genes related to PD.

Gene	Mutation type	DNAchange	Proteinchange	Cases (2N = 498)	Controls (2N = 290)	Sample ID (coverage)
*PARK2*	Missense	c.802G>A^1^	p.Arg234Gln	3	0	Cas74 (58), Cas172(126), Cas214 (75)
(M Recessive)	Frameshift	c.154delA^2^	p.Asn52Metfs	3	1	Cas211 (31), Cas246^ab^ (32), Cas20^ab^(30), Con142(45)
	Exon deletion	Ex3‐4del		3	0	Cas57^c^ (0)^d^, Cas246 (20)^d^
	Exon deletion	Ex2del		2	0	Cas232^c^ (0)^d^
	Missense	c.1244C>A^3^	p.Thr415Asn	1	0	Cas211 (76)
	Missense	c.574A>C^4^	p.Met192Leu	1	0	Cas76 (72)
	Exon deletion	Ex2‐4del		1	0	Cas20 (34)^d^
	Exon deletion	Ex3‐6del		1	0	Cas241 (24)^d^
	Exon duplication	Ex3dup		2	0	Cas148^c^ (149)^d^
	Frameshift	c.220‐221insGT^5^	p.Trp74Cysfs	1	0	Cas11 (29)
	Frameshift	c.101‐102delAG^6^	p.Gln34Argfs	1	0	Cas241^ab^ (25)
*LRRK2* (M Dominant)	Missense	c.6055G>A^7^	p.Gly2019Ser	3	0	Cas213 (126), Cas226 (39), Cas113 (108)
	Stop gain	c.4654C>T^8^	p.Arg1552Ter	1	0	Cas55^a^ (81)
*DJ1*	Exon deletion	Ex4del		1	0	Cas136 (36)^d^
(M Recessive)						
*PINK1*	Stop gain	c.270G>A^8^	p.Trp90Ter	2	0	Cas154^ac^ (2)
(M Recessive)	Stop gain	c.1366C>T^9^	p.Gln456Ter	1	0	Cas194 (30)
*GBA*		RecNcil		1	0	Cas103 (61)^d^

^1^rs144032774 (C/T), ^2^rs754809877 (T/‐), ^3^rs778125254 (G/T), ^4^rs9456735 (T/G), ^5^rs746646126 (‐/CA), ^6^rs55777503 (CT/‐), ^7^rs34637584 (G/A), ^8^Not reported before, ^9^rs45539432 (C/T), ^a^Confirmed with Sanger sequencing, ^b^Hemizygous, ^c^Homozygous, ^d^Average coverage across duplication/deletion. Abbreviations: 2N, number of chromosomes.

### Structural Variants in the *PARK2* Gene

We identified 4 different exon deletions and 1 exon duplication in the *PARK2* gene, affecting a total of 6 PD cases (Figure 1A). All of them have already been described in PD cases.^10^, ^11^ The 2 largest *PARK2* deletions spanned from exon 3 to 6 and from exon 2 to 4 and were found in heterozygosis in Cas241 and Cas20, respectively. These 2 PD cases are examples of compound heterozygotes because both individuals also present different frameshift mutations in exon 2, causing a premature stop codon: Cas241 is heterozygote for a dinucleotide deletion (p.Gln34Argfs), whereas Cas20 is hemizygote for a single‐nucleotide deletion (p.Asn52Metfs; see Table 1, Table S2, and Figure S2). The *PARK2* deletion comprising exons 3 to 4 was found in 2 different PD cases: Cas57, which is homozygote for the deletion, and Cas246, which is an additional *PARK2* compound heterozygote also carrying the p.Asn52Metfs mutation. Finally, Cas232 is found homozygote for a *PARK2* deletion affecting exon 2, and Cas148 is homozygote for a duplication affecting exon 3. As expected, early age at onset (before 45 years of age) was found in 4 of the 6 patients with structural variants in *PARK2*.

### Structural Variants in the *GBA‐PGBA1* Region

The high homology between *GBA* and its neighboring pseudogene (*GBAP1*), which share 96% of sequence identity, not only explains several gene‐pseudogene rearrangements and gene‐conversion events^12^ but also complicates the analysis of the whole region.^13^ Our analysis disclosed 4 individuals presenting CNVs along the *GBA‐GBAP1* region (see Figure 1B). Cas103, which has already been described in Setó‐Salvia et al. (2012),^14^ is heterozygote for a recombinant deletion known as the Rec‐Ncil allele (where *GBAP1* exons 1 to 10 and *GBA* exons 11 and 12 are deleted). In contrast, Cas211, Cas62, and Cas136 are heterozygotes for 2 different duplications along the same region. Because exons 11 and 12 in the gene and the pseudogene are nearly identical, we could not verify by quantitative PCR the exact limits of the *GBA‐GBAP1* rearrangements detected (Figure 1B). Subsequent PCR and sequencing analysis allowed us to confirm that Cas211 is heterozygote for the reciprocal product of the Rec‐Ncil deleted allele (exon 11 and 12 of the *GBA* gene and *GBAP1* exons 1 to 10 are duplicated), whereas Cas62 and Cas136 are heterozygotes for a duplication affecting the 3′unstranslated region of the *GBA* gene and most of the *GBAP1* pseudogene (see details in Figure S3 and Table S3). In addition to the *GBA‐GBAP1* duplication rearrangement, Cas62 also carries the p.Asp370Ser mutation described to increase risk for late‐onset PD^15^ and known to be the most common causal mutation for Gaucher's disease in Ashkenazi Jews^16^ in a heterozygous state (Table S2).

### Structural Variants in the *DJ1* Gene

Among all analyzed participants, only 1 PD patient (Cas136) carried a heterozygous deletion comprising the whole exon 4 of the *DJ1* gene (Figure 1C). As far as we are aware, this alteration has never been reported before.^10^, ^11^ Interestingly, the same individual is also heterozygote for 1 duplication in the *GBA‐GBAP1* region. Therefore, Cas136 could be a particular case of a double heterozygote for genomic rearrangements occurring in 2 different PD loci.

### Other Functional Mutations and Enrichment of Rare Variation

Besides the detected structural variants, other functional alterations (including several known PD Mendelian mutations) had been previously detected in the same dataset.^8^ Frequencies and details are presented in Table 1 and Appendix 4 (Supplemental Data). Here, we further validated particular genotypes by Sanger sequencing in some PD cases to confirm genotypes in regions with low coverage depth as well as novel potential causative variants. Notably, we confirmed the identification of 2 stop‐gain mutations, to our knowledge previously unreported, having checked the PDmutDB,^10^, ^11^ 1000 Genomes Project,^17^ ExAC database (http://exac.broadinstitute.org/, accessed April 26, 2016) and the dbSNP database^18^: p.Arg1552Ter in *LRRK2*, which was found in 1 heterozygote carrier (Cas55), and p.Trp90Ter in *PINK1*, which was found in homozygosis (Cas154) and could also represent a new causal variant for PD (see Figure S2).

In the same dataset, we have previously shown that PD cases displayed significantly higher proportions of rare (minor allele frequency (MAF) <1%) code‐altering (nonsynonymous SNPs, nonsense mutations, and coding indels) variants than controls on Mendelian genes.^8^ Notably, when performing the same type of collapsing analyses, considering only the exon dosage alterations identified here, PD cases also show significant enrichment of CNV in PD Mendelian genes when compared with controls (*P* value <.05 in 10 of 12 tests; Figure S4).

## Discussion

Our analysis demonstrates the usefulness of NGS for discovering different types of variants with a potential role in human disease. Among the detected inactivating variants, we not only report point mutations and small indels but also different exon dosage variants already known to be involved in PD aetiology. Notably, all predicted CNVs were subsequently confirmed by quantitative PCR, suggesting that the analysis of coverage from resequencing data across multiple individuals provides valuable information for the identification of exon dosage variants. It should be noted, however, that the sensitivity of the XHMM software could not be evaluated with this research design and that our study was focused on a limited set of candidate genes. Thus, an unknown number of CNVs could remain undiscovered in this set of patients.

Whereas the analysis of functional SNP variation and indels in Mendelian genes related to PD allowed us to identify putative causative variants for 6 PD cases (Cas211, Cas213, Cas226, Cas113, Cas55, and Cas154), the joint analysis of these inactivating variants, together with the exon dose alterations detected here, probably helps to explain the disease phenotype of 6 additional PD cases (Cas246, Cas20, Cas57, Cas232, Cas148, Cas241) in our Spanish cohort of 249 PD cases (2.4%). Thus, as demonstrated in this dataset, CNVs in the form of exon dose alterations are at least as important as indels and other functional SNP variations in PD Mendelian genes when explaining the phenotype of apparently sporadic PD cases. Given the recognized role of structural variants in several neurodegenerative disorders and because many NGS‐based projects with large numbers of individuals are currently underway to study rare and common variant association, efforts should be made to integrate the analysis of potential CNVs in these new datasets.

## Author Roles

1. Research Project: A. Conception, B. Organization, C. Execution; 2. Statistical Analysis: A. Design, B. Execution, C. Review and Critique; 3. Manuscript Preparation: A. Writing the First Draft, B. Review and Critique.

N.S.: 1A, 1C, 2A, 2B, 3A

A.R‐U.: 1C, 2B, 3A

L.C.: 1B, 1C

M.V.: 1C

R.A.: 1C

J.P.: 1B, 3B

B.P‐S.: 1B, 3B

A.C.: 1B, 3B

J.K.: 1B, 3B

F.C.: 1C, 3B

J.C.: 1A, 1B, 2A, 2C, 3B

E.B.: 1A, 1B, 2A, 2C, 3B

## Full financial disclosure for the previous 12 months

All authors report nothing to disclose.

## Supporting information

Additional Supporting Information may be found in the online version of this article at the publisher's web‐site.

Supporting Information

Supporting Information
