# Supplementary material for: Detection of genomic rearrangements from targeted resequencing data in Parkinson's disease patients
Source: Mov Disord. 2016 Nov 7;32(1):165–9. doi: 10.1002/mds.26845 (PMC5297984; doi:10.1002/mds.26845)

**Appendix 1: subjects and targeted resequencing data**

Targeted resequencing data from 394 individuals, including 249 idiopathic PD cases and 145 unrelated controls of European origin, were compiled from Spataro et al. (2015).^1^ All PD cases fulfilled the diagnostic criteria described by Hughes et al. (1992)^2^ for idiopathic PD and were collected among outpatients regularly attending the Movement Disorders Unit at the Hospital de la Santa Creu i Sant Pau (HSCSP), Barcelona, Spain. In addition, control individuals from the same locality underwent thorough neurological examination and complete neuropsychological assessment to rule out any possible neurological illness; average age at examination was 66.23 ± 8.19 years. See details of clinical and demographic features of PD patients in which structural variants were found in Table A1. Written informed consent approved by the HSCSP ethics committee was obtained from all the individuals participating in the study.

For all subjects, genomic DNA was isolated using a Flexigene DNA kit (Qiagen, Valencia, CA), and quantified with a Nanodrop instrument. As described in Spataro et al. (2015)^1^, after target enrichment with a NimbleGen array and high-throughput sequencing with paired-end reads of 90 bp on a Hiseq2000 platform, we obtained resequencing data for a total of 38 candidate genes previously related to PD (i.e. *RAB25*, *NUCKS1*, *RAB7L1, GBA, SYT11, ACMSD, STK39*, *MCCC1*, *STBD1*, *GAK*, *DGKQ*, *BST1*, *SCARB2*, *HLA-DRB5*, *GPNMB*, *FGF20*, *ITGA8*, *HIP1R*, *STX1B*, *SETD1A*, *SREBF1*, *MED13*, *RAI1*, *MAPT*, *RIT2*, *SNCA*, *LRRK2*, *VPS35*, *PINK1*, *DJ1*, *ATP13A2*, *PARK2*, *FBX07*, *PLA2G6*, *GIGYF2*, *HTRA2*, *EIF4G1*, and *UCHL1*). For each gene, we targeted all possible exons, all potential regulatory sequences overlapping exons as available in Ensemble GRCh37 (http://grch37.ensembl.org/index.html),^3^ plus up to 2,500 bp upstream each gene transcription start site. Original sequence data from Spataro et al. (2015)^1^ is deposited at the European Genome-phenome Archive (EGA, http://www.ebi.ac.uk/ega/), under accession number EGAS00001000973.

**Table A1. Clinical and demographic features of PD patients in which structural variants were found with XHMM.**

| **Gene** | **SV Type** | **Limits of confirmed affected exons by qPCR^a^** | **Genotype** | **Sample ID** | **Gender** | **Age at onset** | **Family history** | **Parkinson type** | **Response to LD** | **Dementia (AO)** |
| --- | --- | --- | --- | --- | --- | --- | --- | --- | --- | --- |
| *PARK2* | Del | Ex2 | Hom | Cas232 | M | 51 | No | Mixed | Yes | No |
|  |  | Ex3-Ex4 | Hom | Cas57 | F | 30 | Yes | Ak-R^h^ | Yes | No |
|  |  | Ex3-Ex4 | C Het^d^ | Cas246 | F | 28 | Yes | Mixed | NA^i^ | No |
|  |  | Ex3-Ex6 | C Het^e^ | Cas241 | M | 28 | No | Ak-R^h^ | NA^i^ | No |
|  |  | Ex2-Ex4 | C Het^d^ | Cas20 | F | 43 | Yes | Mixed | Yes | No |
|  | Dup | Ex3 | Hom | Cas148 | M | 69 | Yes | Mixed | Not treated | No |
| *GBA-GBAP1* | Del | Ex10 *GBAP1-*Down Ex12^b^ *GBA* | Het | Cas103^f^ | M | 51 | No | Mixed | Yes | No |
|  | Dup | Ex10 *GBAP1-*Down Ex12^b^ *GBA* | Het | Cas211^d,g^ | F | 37 | No | Mixed | Yes | No |
|  |  | Ex10 *GBAP1-*Down Ex12 *GBA*^c^ | Het | Cas62 | F | 52 | No | Mixed | Yes | Yes (62) |
|  |  | Ex10 *GBAP1-*Down Ex12 *GBA*^c^ | Het | Cas136 | F | 52 | Yes | Mixed | Yes | Yes (76) |
| *DJ1* | Del | Ex4 | Het | Cas136 | F | 52 | Yes | Mixed | Yes | Yes (76) |

^a^ Confirmed exons by qPCR analysis (see Table S4 for correspondence with exon Ensembl IDs), ^b^PCR and sequencing analysis extends del/dup to Ex11 *GBA*, ^c^PCR and sequencing analysis extends duplication from Ex11 *GBAP1* to the 3’UTR of the *GBA* gene , ^d^p.Asn52Metfs (rs754809877), ^e^p.Gln34Argfs (rs55777503), ^f^ RecNcil/wt, ^g^ p.Thr415Asn (rs778125254), ^h^Akinetic-rigid syndrome, ^i^ Not available. Abbreviations: SV, structural variant; Del, deletion; Dup, duplication; LD, levodopa; AO, Age at Onset (years); Hom, homozygous, C Het, compound heterozygous, Het, heterozygous

Appendix 2: real-time PCR design and copy number analysis

Primers and probes for quantitative PCR analysis were designed with the Primer Express 3.01 software (Life Technologies) using sequences available in Ensembl GRCh37 (http://grch37.ensembl.org/index.html) for *DJ-1* (ENSG00000116288), *PARK2* (ENSG00000185345), *GBA* (ENSG00000177628) and *GBAP1* (ENSG00000160766). Genomic sequences around the *GBA* gene and the *GBAP1* pseudogene were aligned using ClustalW (http://www.ebi.ac.uk/Tools/msa/clustalo)^4^ to ensure that *GBA* and *GBAP1* primers and probes were specific for their intended targets. Probes were tagged with FAM as a reporter and with either MGB or TAMRA as a quencher at the 3’ end. Sequences for all primer and probes for qPCR analysis and details on quencher dyes are available in Table S4. Primers and probes were synthesized by Sigma (Life Science) and Life Technologies (currently Thermo Fisher Scientific), respectively.

Reactions were set up with 12µM of each primer, 2.5 µM Copy Number Custom TaqMan probe, 1X TaqMan Gene Expression MasterMix, 1X TaqMan Copy Number Reference Assay RNase P tagged with VIC (Catalog number 4403326) and 32 ng DNA in a final volume of 5 uL. All samples were assayed for each corresponding exon in quadruplicate in 384-well plates in a QuantStudio™ 12K Flex Real-Time PCR System (Life Technologies). The thermal conditions were 50°C for 2 min, 95°C for 10 min and 40 cycles of 15 s at 95°C, and 60°C for 1 min. Finally, the exon copy number was determined using the ExpressionSuite Software (Thermo Fisher Scientific) and the 2(-ΔΔCt) method.^5^

Appendix 3: PCR and Sanger sequencing validation analysis

Amplification and sequencing primers were designed with Primer3 (http://bioinfo.ut.ee/primer3/).^6^ Primer sequences and thermal conditions for amplification and sequencing are available in Table S5. PCR amplifications around frameshift deletions and stop gains were set up with 0.5 µM of each primer, 0.8 mM of dNTPs (0.2 mM each), 3 mM of MgCl_2_, 1X of NH4 Reaction Buffer, 1U BioTaq (Bioline) and 25 ng of DNA in a final volume of 25 uL. PCR reactions to amplify selected fragments around the *GBA-GBAP1* region were modified to include 2.5 mM of MgCl_2_. PCR products were purified using the Illustra GFX PCR DNA and Gel Band Purification Kit (GE Healthcare Life Sciences) following the manufacturer’s recommendations.

PCR sequencing reactions were performed in a final volume of 10µL containing 5 ng of purified PCR product, 1X DNA Sequencing Buffer, 0.25µL of BigDye® Terminator v3.1, and 3.2 µM of primer. All PCR reactions were performed on an Applied Biosystems GeneAmp PCR System 9700. Sequencing products were purified using the HighPrep™ DTR BigDye Sequencing Clean Up kit (MAGBIO) following standard manufacturers’ recommendations and run on a 96-capillary 3730xl ABI sequencer at the Genomics Unit of the Universitat Pompeu Fabra. Sequence analysis and visualization were performed with either the Seqman module of the DNASTAR Lasergene software ver. 7.1.0 (DNASTAR, Inc. Madison, WI U.S.) or the Sequence Scanner Software 2 (Applied Biosystems).

**Appendix 4: other functional mutations in the dataset**

Within the *PARK2* gene, in addition to the frameshift indels reported in the main text, we detected three heterozygous carriers for the PD recessive mutation p.Arg234Gln (Cas74, Cas172, Cas214), one heterozygous carrier for the PD recessive mutation p.Met192Leu (Cas76) and one additional compound heterozygote carrying the frameshift p.Asn52Metfs and the PD recessive mutation p.Thr415Asn (Cas211)^1^. Only one control individual (Con142) was heterozygote for the p.Asn52Metfs frameshift in *PARK2*. As for *LRRK2*, we not only detected three heterozygotes for the PD dominant mutation p.Gly2019Ser (Cas213, Cas226, Cas113) but also one heterozygote carrier (Cas55) for a stop-gain mutation (p.Arg1552Ter), to our knowledge not previously reported, having checked in the PDmutDB^7,8^, 1000 Genomes Project^9^, ExAC database (http://exac.broadinstitute.org/, accessed 26/04/2016) and the dbSNP database.^10^ Finally, in *PINK1* we detected one heterozygote carrier for the stop-gain p.Gln456Ter (Cas194) plus one homozygote (Cas154) for a new stop-gain (p.Trp90Ter), which could also represent a new causal variant for PD.

**References**

1. Spataro N, Calafell F, Cervera-Carles L, Casals F, Pagonabarraga J, Pascual-Sedano B, et al. Mendelian genes for Parkinson’s disease contribute to the sporadic forms of the disease. Hum Mol Genet. 2014;24(7):2023–34. Available from: <http://www.hmg.oxfordjournals.org/cgi/doi/10.1093/hmg/ddu616>

2. Hughes AJ, Daniel SE, Kilford L, Lees AJ. Accuracy of clinical diagnosis of idiopathic Parkinson’s disease: a clinico-pathological study of 100 cases. J Neurol Neurosurg Psychiatry. 1992 Mar;55(3):181–4. Available from: <http://www.ncbi.nlm.nih.gov/pmc/articles/PMC1014720/>

3. Flicek P, Amode MR, Barrell D, Beal K, Billis K, Brent S, et al. Ensembl 2014. Nucleic Acids Res. 2014;42(D1):749–55.

4. McWilliam H, Li W, Uludag M, Squizzato S, Park YM, Buso N, et al. Analysis Tool Web Services from the EMBL-EBI. Nucleic Acids Res. 2013;41(Web Server issue):597–600.

5. Livak KJ, Schmittgen TD. Analysis of Relative Gene Expression Data Using Real-Time Quantitative PCR and the 2−ΔΔCT Method. Methods. 2001 Dec;25(4):402–8. Available from: <http://www.sciencedirect.com/science/article/pii/S1046202301912629>

6. Untergasser A, Cutcutache I, Koressaar T, Ye J, Faircloth BC, Remm M, et al. Primer3—new capabilities and interfaces. Nucleic Acids Res. 2012 Aug 1;40 (15 ):e115–e115. Available from: <http://nar.oxfordjournals.org/content/40/15/e115.abstract>

7. Nuytemans K, Theuns J, Cruts M, Van Broeckhoven C. Genetic etiology of Parkinson disease associated with mutations in the *SNCA, PARK2, PINK1, PARK7*, and *LRRK2* genes: a mutation update. Hum Mutat. 2010 Jul 1;31(7):763–80. Available from: <http://dx.doi.org/10.1002/humu.21277>

8. Cruts M, Theuns J, Van Broeckhoven C. Locus-specific mutation databases for neurodegenerative brain diseases. Hum Mutat. 2012 Sep 1;33(9):1340–4. Available from: <http://dx.doi.org/10.1002/humu.22117>

9. The 1000 Genomes Project Consortium. An integrated map of genetic variation from 1,092 human genomes. Nature. 2012;135(V):0–9.

10. Sherry ST, Ward M-H, Kholodov M, Baker J, Phan L, Smigielski EM, et al. dbSNP: the NCBI database of genetic variation. Nucleic Acids Res. 2001 Jan 1;29 (1 ):308–11. Available from: http://nar.oxfordjournals.org/content/29/1/308.abstract

**Supplementary Figure S1. Validation of copy number variation within the *PARK2* gene (A), the *GBA-GBAP1* region (B) and the *DJ1*gene (C).** Relative quantification (RQ) values for each targeted exon (y axis) and sample. A standard deviation of 1.96 was selected in order to determine the RQ minimum and maximum values (error bars) for each sample.

**A**

**
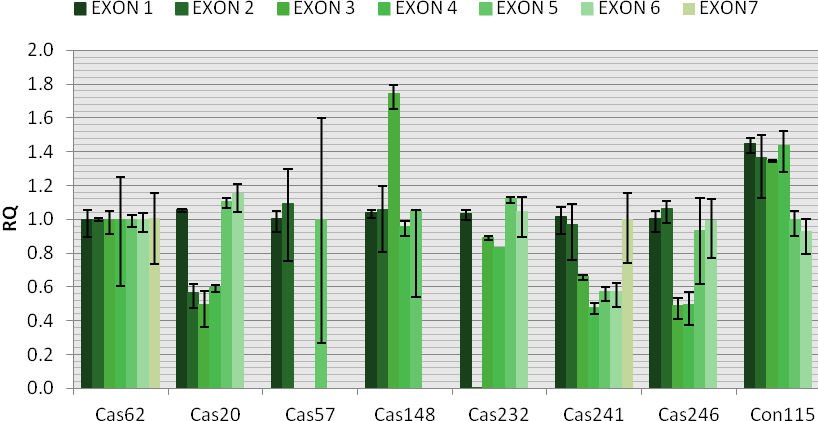
**

**B**

**
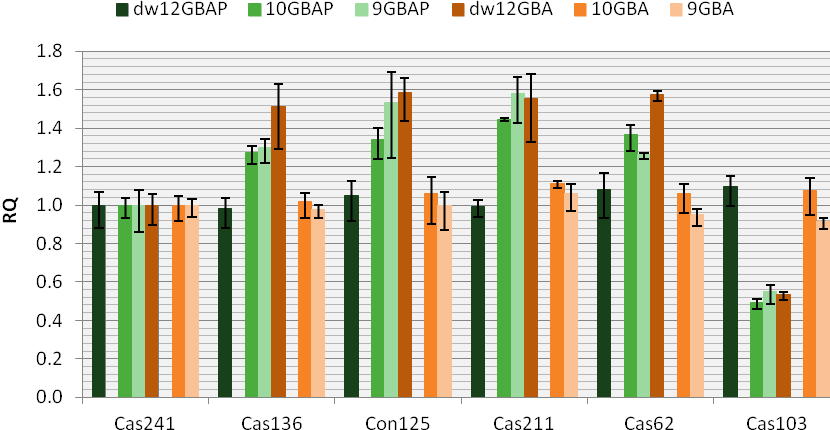
**

**C**

**
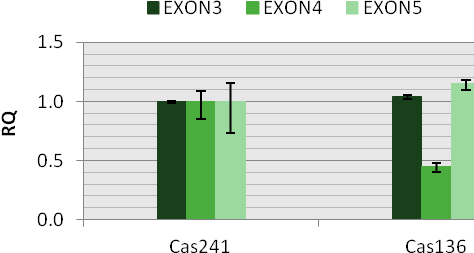
**

**Supplementary Figure S2. (A) Frameshift indels detected in individuals heterozygous for different exon deletions at *PARK2*.** Sequence chromatograms showing the location of detected frameshifts in Cas241 (heterozygote), Cas20 (hemizygous) and Cas246 (heterozygote). **(B) New stop gain detected at *LRRK2*.** Sequence chromatograms showing the location of the detected stop gain in Cas55 (heterozygote). (C) **New stop gain detected at *PINK1*.** Sequence chromatograms showing the location of the detected stop gain in Cas154 (homozygote).

**A**


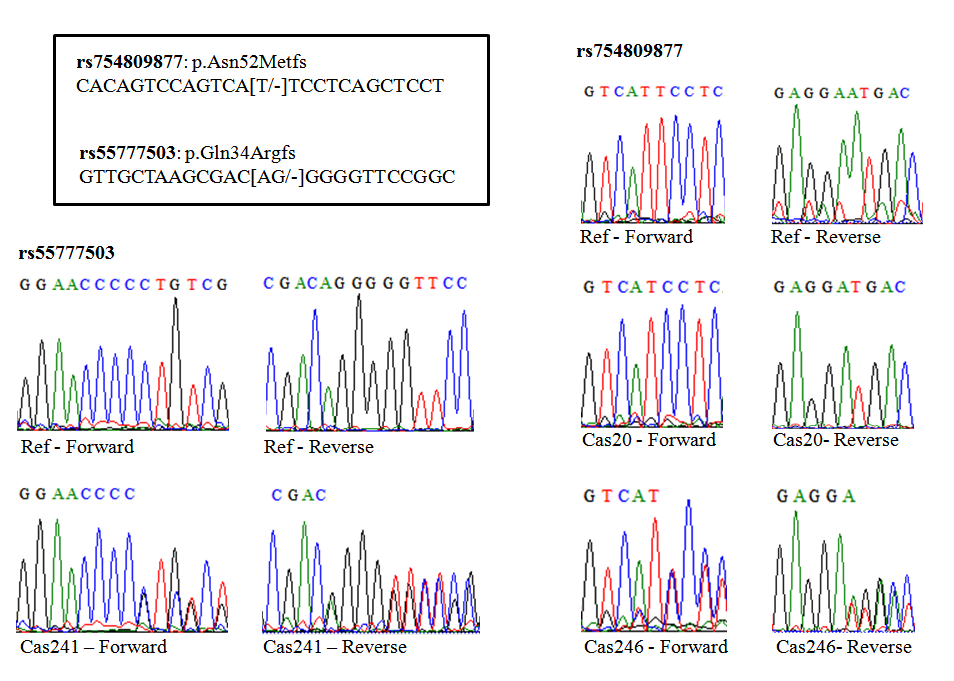


**B**


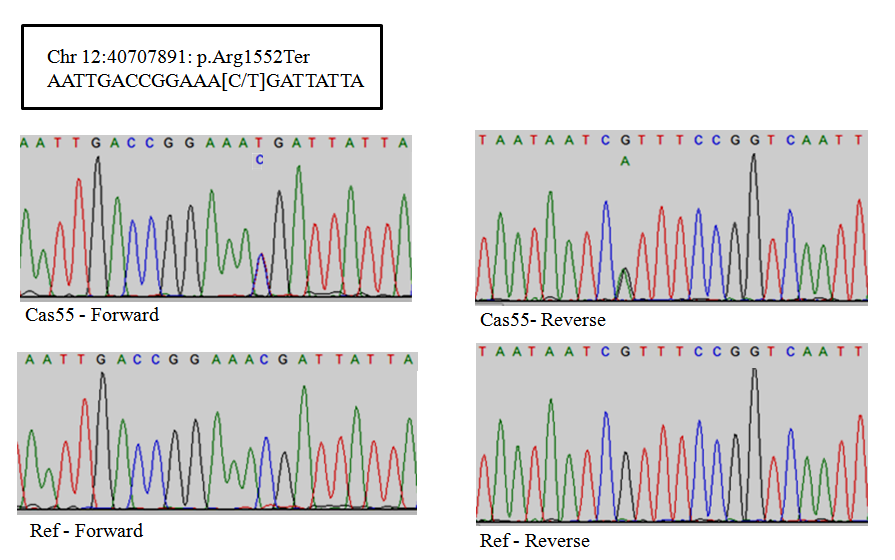


**C**


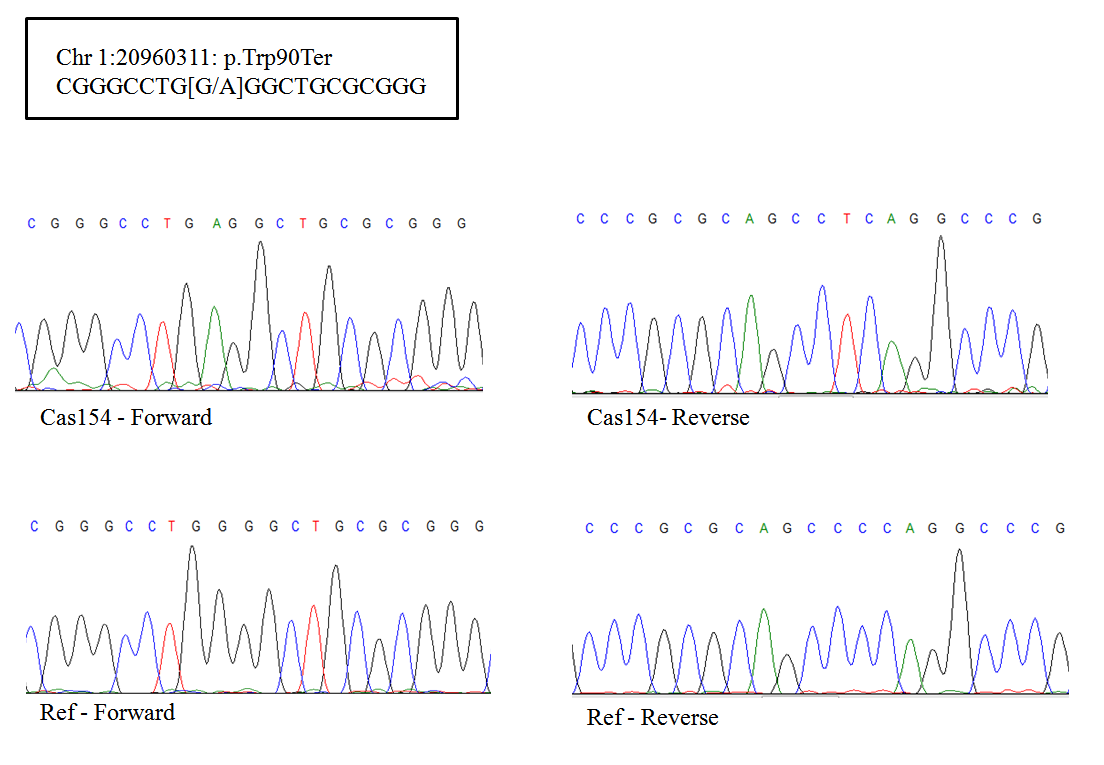


**Supplementary Figure S3. Schematic representation of genomic rearrangements detected around the *GBA-GBAP1* region.** (A) Cas103 is heterozygote for a recombinant deletion known as the Rec-Ncil allele, where *GBAP1* exons 1 to 10 and *GBA* exons 11 and 12 are deleted. (B) Cas211 is heterozygote for the reciprocal product of the Rec-Ncil deleted allele (exons 11 and 12 of the *GBA* gene and exons 1 to 10 of the *GBAP1* are duplicated). (C) Cas62, Con125 and Cas136 are heterozygotes for a duplication affecting the 3’UTR region of the *GBA* gene and most of the *GBAP1* pseudogene*.*


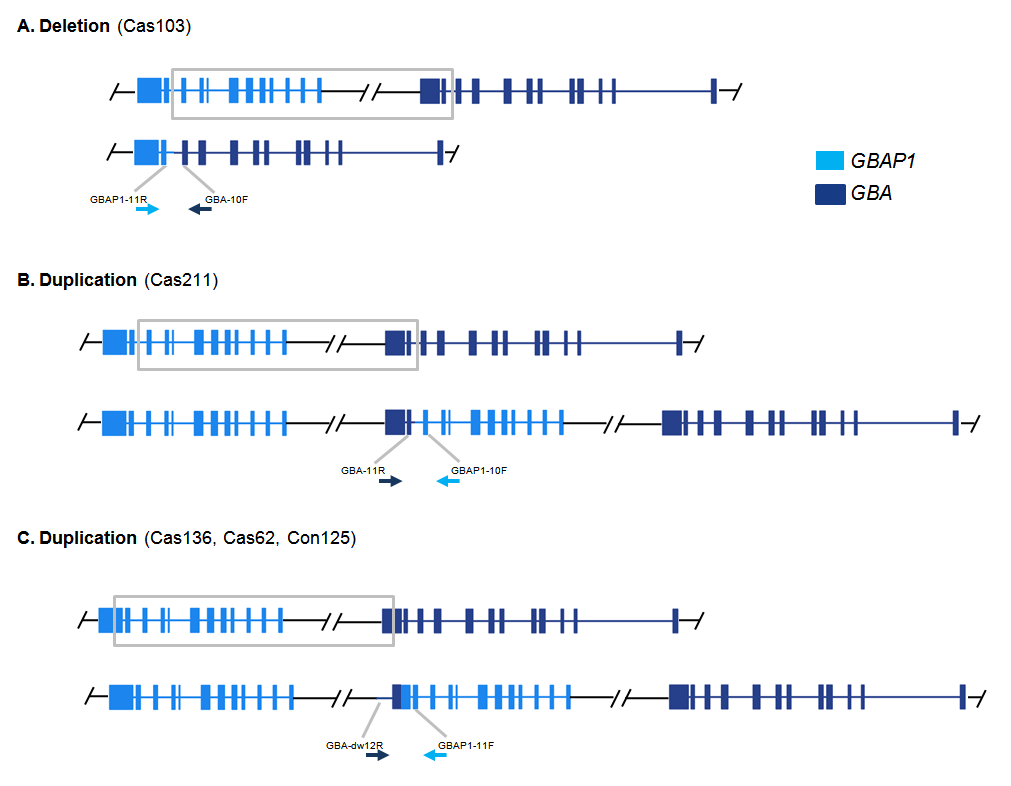


**Supplementary Figure S4. Statistical significances in several collapsing tests**. A number of association tests for rare CNVs were performed using the Variant Association Tools options within the Variant Tools software (http://varianttools.sourceforge.net/). ASUM, data-adaptive Sum test; CMC, Combined and Multivariate Collapsing test; KBAC, Kernel Based Adaptive Clustering; RTB, Replication Based Test; VT, Variable Thresholds method; WSS, Weighted Sum Statistic.


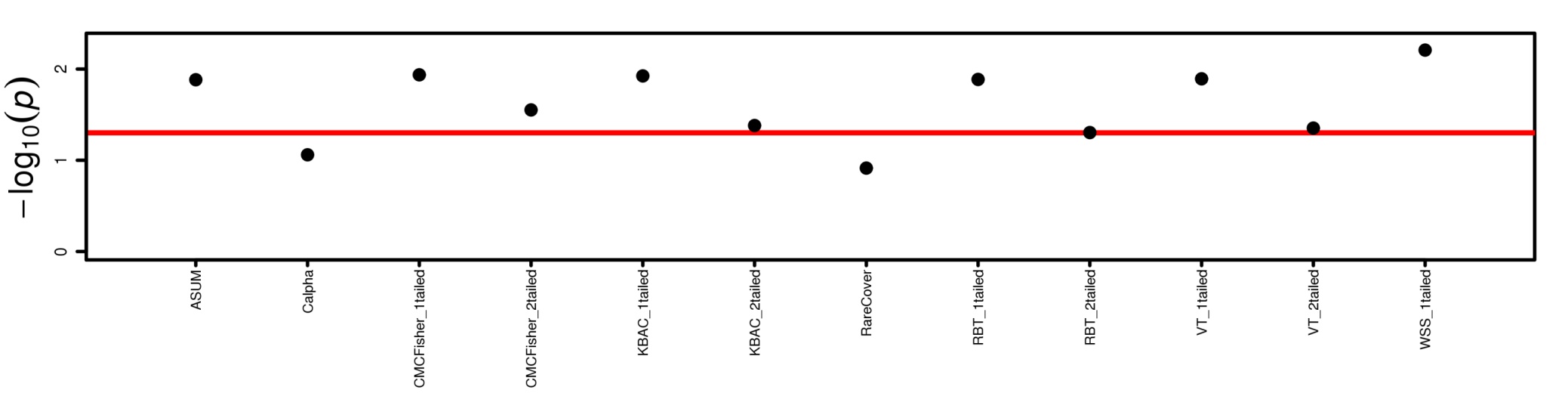

Supplement: Supplementary file 1 — Supporting Information [file MDS-32-165-s001.doc]
