# Supplementary material for: Detection of genomic rearrangements from targeted resequencing data in Parkinson's disease patients
Source: Mov Disord. 2016 Nov 7;32(1):165–9. doi: 10.1002/mds.26845 (PMC5297984; doi:10.1002/mds.26845)
Supplement: Supplementary file 2 — Supporting Information [file MDS-32-165-s002.pdf]

Q< 10 (>10% error)
10<Q<20 (10-1% error)

Table S1. CNVs detected with the XHMM software.

Sample Code	Gene	Predicted Region with the XHMM software	CNV	Kb	Q_EXACT	Q_SOME	Q_NON_DIPLOID	Q_START	Q_STOP	MEAN_RD	Limits of confirmed affected exons[a]	Genotype
Cas136	*DJ1*	Chr1: 8029096 - 8029514	Del	0.42	57	57	57	32	10	-7.07	Ex4	Het
Cas103[f]	*GBA* - *GBAP1*	Chr1:155184846 - 155205685	Del	20.83	3	99	99	38	3	-4	Ex10 *GBAP1*- Down Ex12[b] *GBA*	Het
Cas136	*GBA* - *GBAP1*	Chr1: 155184360 - 155203638	Dup	19.28	8	99	99	24	8	3.81	Ex10 *GBAP1*- Down Ex12 *GBA* [c]	Het
Cas211	*GBA* - *GBAP1*	Chr1:155184846 - 155203638	Dup	18.79	12	99	99	26	4	4.08	Ex10 *GBAP1*- Down Ex12[b] *GBA*	Het
Cas62	*GBA* - *GBAP1*	Chr1:155184360 - 155203638	Dup	19.28	16	99	99	22	16	4	Ex10 *GBAP1*- Down Ex12 *GBA* [c]	Het
Con125	*GBA* - *GBAP1*	Chr1:155184360 - 155203638	Dup	19.28	12	99	99	19	12	3.96	Ex10 *GBAP1*- Down Ex12 *GBA* [c]	Het
Cas148	*PARK2*	Chr6:162683444 - 162683847	Dup	0.40	30	30	30	29	30	10	Ex3	Hom
Cas232	*PARK2*	Chr6:162864293 - 162864559	Del	0.27	31	31	31	29	29	-10	Ex2	Hom
Cas20	*PARK2*	Chr6:162622114 - 162864559	Del	242.45	79	99	99	37	33	-7.98	Ex2-Ex4	C Het[d]
Cas241	*PARK2*	Chr6:162394285 - 162683847	Del	289.56	54	99	99	51	32	-6.6	Ex3-Ex6	C Het[e]
Cas246	*PARK2*	Chr6:162622114 - 162683847	Del	61.73	66	74	74	17	49	-7.57	Ex3-Ex4	C Het[d]
Cas57	*PARK2*	Chr6:162622114 - 162683847	Del	61.73	97	99	99	37	46	-9.59	Ex3-Ex4	Hom
Con115	*PARK2*	Chr6:162622114 - 163151303	Dup	529.19	35	99	99	22	35	6.49	Ex1-Ex4	Het

[a] Confirmed exons by qPCR analysis (see Table S2 for correspondence with exon Ensembl IDs)

[b] PCR and sequencing analysis extends deletion/duplicaton to Ex11 *GBA* , [c] PCR and sequencing analysis extends duplication from Ex11 *GBAP1* to the 3'UTR of the *GBA* gene, [d]rs754809877 (p.Asn52Metfs), [e]rs55777503 (p.Gln34Argfs), [f]Already known as RecNcil/wt

Abbreviations:

Q-EXACT, Phred-scaled quality of the exact CNV event along the entire interval; Q_SOME, Phred-scaled quality of some CNV event in the interval; Q_NON_DIPLOID, Phred-scaled quality of not being diploid; Q_START, Phred-scaled quality of "left" breakpoint of CNV; Q_STOP, Phred-scaled quality of "right" breakpoint of CNV; MEAN_RD, Mean normalized read depth (z-score) over interval

Hom, Homozygote; Het, heterozygote; C Het, Compound heterozygote

Table S2. Detailed genotypes and clinical features of PD samples in which potential functional variants were found

Gene	Sample ID	Genotypes	Gender	Age at onset	Family history	UPDRS	H&Y	Parkinson type	Response to LD	Dementia (AO)
PARK2	Cas232	Ex2del<br>Ex2del	M	51	No	27	2	Mixed	Yes	No
	Cas57	Ex3-4del<br>Ex3-4del	F	30	Yes	17	2	Ak-R	Yes	No
	Cas246	Ex3-4del<br>p.Asn52Metfs	F	28	Yes	23	3	Mixed	NA	No
	Cas241	Ex3-6del<br>p.Gln34Argfs	M	28	No	31	2.5	Ak-R	NA	No
	Cas20	Ex2-4del<br>p.Asn52Metfs	F	43	Yes	16	3.5	Mixed	Yes	No
	Cas211[a]	p.Asn52Metfs<br>p.Thr415Asn	F	37	No	18	2	Mixed	Yes	No
	Cas74	p.Arg234Gln<br>wt	M	48	Yes	27	5	Ak-R	Yes	Yes (61)
	Cas172	p.Arg234Gln<br>wt	F	50	No	17	2	Ak-R	Yes	No
	Cas214	p.Arg234Gln<br>wt	M	NA	Yes	22	2	Pred Trem	Yes	NA
	Cas76	p.Met192Leu<br>wt	F	60	No	42	5	Ak-R	Yes	No
	Cas11	p.Trp74Cysfs<br>wt	M	38	No	36	2	Pred Trem	Yes	No
	Cas148	Ex3dup<br>Ex3dup	M	69	Yes	20	2	Mixed	Not treated	No
LRRK2	Cas213	p.Gly2019Ser<br>wt	M	50	Yes	21	3	Ak-R	Yes	NA
	Cas226	p.Gly2019Ser<br>wt	NA	NA	NA	NA	NA	NA	NA	NA
	Cas113	p.Gly2019Ser<br>wt	M	56	Yes	34	2.5	Pred Trem	Yes	No
	Cas55	p.Arg1552Ter<br>wt	M	33	No	23	2.5	Mixed	Yes	No
PINK1	Cas154	p.Trp90Ter<br>p.Trp90Ter	F	40	Yes	8	1	Ak-R	Yes	No
	Cas194	p.Gln456Ter<br>wt	M	32	Yes	15	1.5	Pred Trem	Not treated	NA
DJ1	Cas136[b]	Ex4del<br>wt	F	52	Yes	59	5	Mixed	Yes	Yes (76)
GBA-GBAP1	Cas136[b]	Ex10 *PGBA* -Dw Ex12 *GBA* dup<br>wt	F	52	Yes	59	5	Mixed	Yes	Yes (76)
	Cas211[a]	Ex10 *PGBA* -Dw Ex12 *GBA* dup<br>wt	F	37	No	18	2	Mixed	Yes	No
	Cas62	Ex10 *PGBA* -Dw Ex12 *GBA* dup<br>wt<br>p.Asp370Ser<br>wt	F	52	No	49	4	Mixed	Yes	Yes (62)
	Cas103	RecNcil del<br>wt	M	51	No	12	2	Mixed	Yes	No

[a] Note that this individual carries mutations both at *PARK2* and *GBA-GBAP1* ; [b] Note that this individual carries mutations both at *DJ1* and *GBA-GBAP1*

Abbreviations: NA, not available; wt, wildtype; Ak-R, Akinetic-rigidsyndrome; Pred Trem, tremor dominant

Table S3. Schematic representation of polymorphic positions detected and sequencing analysis of recombinant alleles.

	Amp 11RP-10FP (*GBAP1*)							
	Seq GBA1011R		Seq GBA1011F					
	93-95	70	23	151	184	311	346	360
**GRCh37 Chr1**	155184913-155184914	155184891	155184704	155184576	155184543	155184416	155184381	155184367
Cas103	del55	CC	GG	TT	CC	CC	CC	CC
Con125	del55	CC	GG	TT	CC	CC	CC	NN
Cas136	del55	CC	GG	TT	CC	CC	CC	CC
Cas211	del55	CC	GG	TT	CC	CC	CC	CC
Cas62	del55	CC	GG	TT	CC	CC	CC	CC

	Ampl dw12RP-11FP (*GBAP1*)													
	Seq 11FP				Seq GBA11dw12F									
	23	246	256	319	103	130	191	193	199	201	211	221	225	228
**GRCh37 Chr1**	155184290-155184000	155184067	155184057	155183994	155182803	155182776	155182716-155182715	155182714	155182708	155182706	155182696	155182686	155182682	155182679
Cas103	-	AG	TC	TC	TT	CC	--	AA	TT	AA	TT	TT	AA	CC
Con125	InsA	AA	TC	TT	CT	TC	A/-							
Cas136	-	AA	TC	TT	CT	TC	A/-							
Cas211	-	AA	TC	TT	TT	CC	--	AA	TT	AA	TT	TT	AA	CC
Cas62	-	AA	TC	TT	CT	TC	A/-							

	Amp 11RG-10FG (*GBA*)							
	Seq GBA1011R		Seq GBA1011F					
	93-95	70	23	151	184	311	346	360
**GRCh37 Chr1**	155205595-155205540	155205518	155205331	155205203	155205170	155205043	155205008	155204994
Cas103	ins55	GG	GG	CC	TT	TT	GG	GG
Con125	ins55	GG	AG	CC	TT	TT	GG	GG
Cas136	ins55	GG	GG	CC	TT	TT	GG	NN
Cas211	ins55	GG	AA	CC	TT	TT	GG	GG
Cas62	ins55	GG	AG	CC	TT	TT	GG	GG

	Ampl dw12RG-11FG (*GBA*)													
	Seq 11FG				Seq GBA11dw12F									
	23	246	256	319	103	130	191	193	199	201	211	221	225	228
**GRCh37 Chr1**	155204917-155204916	155204694	155204684	155204621	155203430	155203403	155203342	155203340	155203334	155203332	155203322	155203312	155203308	155203305
Cas103	-	GG	TT	CC	CC	TT	AA	GG	CC	CC	CC	AA	GG	TT
Con125	-	GG	TT	TT	CC	TT	AA	GG	CC	CC	CC	AA	GG	TT
Cas136	-	GG	TT	TT	CC	TT	AA	GG	CC	CC	CC	AA	GG	TT
Cas211	-	GG	TT	TT	CC	TT	AA	GG	CC	CC	CC	AA	GG	TT
Cas62	-	GG	TT	TT	CC	TT	AA	GG	CC	CC	CC	AA	GG	TT

	Amp 10FG-11RP (deletion)							
	Seq GBA1011R		Seq GBA1011F					
	93-95	70	23	151	184	311	346	360
**GRCh37 Chr1**	155205595-155205540	155205518	155184704	155184576	155184543	155184416	155184381	155184367
Cas103	ins55	GG	GG	TT	CC	CC	CC	CC

	Ampl dw12RG-11FP (duplication)													
	Seq 11FP				Seq GBA11dw12F									
	23	246	256	319	103	130	191	193	199	201	211	221	225	228
**GRCh37 Chr1**	155184290-155184000	155184067	155204684	155204621	155203430	155203403	155203342	155203340	155203334	155203332	155203322	155203312	155203308	155203305
Con125	-	AA	TT	TT	CC	TT	AA	GG	CC	CC	CC	AA	GG	TT
Cas136	-	AA	TT	TT	CC	TT	AA	GG	CC	CC	CC	AA	GG	TT
Cas62	-	AA	TT	TT	CC	TT	AA	GG	CC	CC	CC	AA	GG	TT

	Amp 10FP-11RG (duplication)							
	Seq GBA1011R		Seq GBA1011F					
	93-95	70	23	151	184	311	346	360
**GRCh37 Chr1**	155184913-155184914	1.55E+08	1.55E+08	1.55E+08	1.55E+08	1.55E+08	1.55E+08	1.55E+08
Cas211	del55	CC	GG	CC	TT	TT	GG	GG

Note: PCR amplifications were performed for two regions within the *GBAP1* pseudogene (with primer pairs 11RP-10FP and 12RP-11FP) and the *GBA* gene (with primer pairs 11RG-10FG and 12RG-11FG), respectively. Recombinant products were obtained for Cas103 (with primer pair 10FG-11RP), Cas211 (with primer pair 10FP-11RG), Cas136, Cas62 and Con125 (with primer pair dw12RG-11FG). In light blue, alleles typical of the *GBAP1* pseudogene; in dark blue, alleles typical of the *GBA* gene. Positions 311, 346 and 360 obtained with the GBA1011F sequencing primer correspond to the Leu444Pro (c.1448T>C), Ala456Pro (c.1483G>C) and Val460Val (c.1497G>C) GBA mutations. Underlined alleles correspond to positions included in one of the amplification primers.

Table S4. Probes and primers for analysis of copy number variation

Gene	Exon/Introns[a]	*Probe/Primer*	Sequence
DJ-1	Ex3 (ENSE00003623984)	Probe	FAM-CTGATGCCAGCCTTG -MGB
		Forward (5'-3')	GTAGCCGTGATGTGGTCATTTG
		Reverse (5'-3')	AAGTCCTCACCCTCTTAATCTGTCA
DJ-1	Ex4 (ENSE00003584479)	Probe	FAM-CAGGAGGTAATCTGGGC-MGB
		Forward (5'-3')	GTTTTAAACTGTTACAGGGACCATATGA
		Reverse (5'-3')	CACAGCCTCCTCCCGAAATA
DJ-1	Ex5 (ENSE00003499981)	Probe	FAM-TTTTCACTAGTCTGCTGCTG-MGB
		Forward (5'-3')	GTGATTGGTTAGTGGCTTAATGATAACT
		Reverse (5'-3')	GAATCAAACCATCGAATGAAAGG
GBA	Intron 8-9	Probe	FAM-TTCCCGTCACCCAACT-MGB
		Forward (5'-3')	TCCCCAAACCTCTCTAGTTGCAT
		Reverse (5'-3')	GCACACAGGCTTCTGGAACTTC
GBA	Ex10 (ENSE00003506590)	Probe	FAM- ACAGTCCCATCATTGTAG -MGB
		Forward (5'-3')	CCAATTGGGTGCGTAACTTTG
		Reverse (5'-3')	TGTAAAACGTGTCCTTGGTGATG
GBA	Down* Ex12 (ENS00001917720)	Probe	FAM- CCCACGCACAGCC -MGB
		Forward (5'-3')	CCCTACCCTTTTGTACCTCTGAGA
		Reverse (5'-3')	AGATACTGGCCCTGGTGACAGT
GBAP1	Intron 8-9	Probe	FAM- CACCTAATTCAGGATTCCTA -MGB
		Forward (5'-3')	TCCCAAAACCTCTGTAGTTGCAT
		Reverse (5'-3')	CCACAGGCTTCTGGAACTTCTAG
GBAP1	Ex10 (ENSE00003507976)	Probe	FAM- AGACATCACCAAGCAC -MGB
		Forward (5'-3')	GACCGACTGGAACCCATCATT
		Reverse (5'-3')	GAACATGGGCTGTTTGTAAAACG
GBAP1	Down* Ex12	Probe	FAM- CCCCACACAGTCACACT -MGB
		Forward (5'-3')	CCCTACCCTTTTGTACCTCTGAGA
		Reverse (5'-3')	GGAGGTCCCTGGGATAGAAACT
PARK2	Ex1 (ENSE00001442937)	Probe	FAM-TACGTGGGTACCTGCC-MGB
		Forward (5'-3')	CCACCTACCCAGTGACCATGA
		Reverse (5'-3')	CGGCGCAGAGAGGCTGTA
PARK2	Ex2 (ENSE00003536181)	Probe	FAM-CCAGCATCTTCCAGCTCAAGGAGGTG-TAMRA
		Forward (5'-3')	CCCAGTGGAGGTCGATTCTG
		Reverse (5'-3')	CCCCCTGTCGCTTAGCAA
PARK2	Ex3 (ENSE00003487128)	Probe	FAM-TGACCTGGATCAGCAGAGCATTGTTCA-TAMRA
		Forward (5'-3')	TTTACCTTGCTCCCAAACAGAATT
		Reverse (5'-3')	TCTCCACGGTCTCTGCACAA
PARK2	Ex4 (ENSE00003576586)	Probe	FAM-TTTTATGTGTATTGCAAAGGCCCCTGTCA-TAMRA
		Forward (5'-3')	TTCTTCTCCAGCAGGTAGATCAATC
		Reverse (5'-3')	TTTTCCCGGCTGCACTCTT
PARK2	Ex5 (ENSE00003540557)	Probe	FAM-CCACACTGCCCTGGGACTAGTGCA-TAMRA
		Forward (5'-3')	CCGGATGAGTGGTGAATGC
		Reverse (5'-3')	AGAGGAATGAATGTGACCAGGTACT
PARK2	Ex6 (ENSE00002151207)	Probe	FAM-AAACATCAGTAGCTTTGCACCTGATCGCA-TAMRA
		Forward (5'-3')	GCACACCCCACCTCTGACA
		Reverse (5'-3')	TGCAAGTGATGTTCCGACTATTTG
PARK2	Ex7 (ENSE00002169394)	Probe	FAM-ACTGTTTCCACTTATACTGTG-MGB
		Forward (5'-3')	CCGCCACGTGATTTGCTTA
		Reverse (5'-3')	CTGCCGATCATTGAGTCTTGTC
PARK2	Ex8 (ENSE00001303667)	Probe	FAM-CAGGCAGCGGCCA-MGB
		Forward (5'-3')	TTCTAAAGAGGTGCGGTTGGA
		Reverse (5'-3')	AAAAAGCCAAACCCTCAAAAGAG

* Downstream exon 12

[a] Ensembl exon ID

Table S5. Primers for sequencing analysis

Gene	Region	Primer Sequencing Analysis	Sequence	Ampl conditions	Seq conditions
PARK2	Exon 2	Ampl/Seq Forward (5'-3')	CAGTGTGGAGTAAAGTTCAAGGA	94°C for 2 min; 32 cycles of 1 min at 94°C, 1 min at 56ºC (or modified according to Ta) and 72°C for 1 min and 30 s; 5 min at 72ºC	94°C for 3 min; 40 cycles of 10 s at 96°C, 5 s at 50ºC (or modified according to Ta) and 60°C for 4 min
	(Gln34, Asn52)	Amplification Reverse (5'-3')	GAGGGGTAAATCGGTTGAGAA		
		Internal Reverse (5'-3')	TGCTCACTTTCTCTTCTCCCT		
	Arg1552F	Ampl/Seq Forward (5'-3')	TTTTTACGGCTTGTCATTTG		
	Arg1552R	Ampl/Seq Reverse (5'-3')	CACTCAGGGACTCTTTCCAC		
PINK	Trp90AF	Ampl/Seq Forward (5'-3')	CTAACCGTCTCCGCTTCTTC		
	Trp90AR	Ampl/Seq Reverse (5'-3')	ACTAATCCTTTGGTTAAACGGC		
GBA	Exon 10	Amplification Forward (5'-3')	CCAATTGGGTGCGTAACTTTG	94°C for 2 min; 32 cycles of 1 min at 94°C, 1 min at 62ºC (or modified according to Ta) and 72°C for 2 min and 30 s; 5 min at 72ºC	94°C for 3 min; 40 cycles of 10 s at 96°C, 5 s at 50ºC (or modified according to Ta) and 60°C for 4 min
	Exon 11	Internal Forward (5'-3')	GCTCTGCTGTTGTGGTCGTG		
		Internal Reverse (5'-3')	TTTAGCACGACCACAACAGC		
	dwExon 12*	Amplification Reverse (5'-3')	TGAGACAGATACTGGCCCTG		
GBAP1	Exon 10	Amplification Forward (5'-3')	GACCGACTGGAACCCATCATT		
	Exon 11	Internal Forward (5'-3')	GCTCTCCTGTTGTGGTCGTC		
		Internal Reverse (5'-3')	TTTAGGACGACCACAACAGG		
	dwExon 12*	Amplification Reverse (5'-3')	GGAGGTCCCTGGGATAGAAACT		
GBA/GBAP1	1011	Internal Forward (5'-3')	TCGGATGGCTTACATCACTCT		
	1011	Internal Reverse (5'-3')	AGAGTGATGTAAGCCATCCGA		
	11dw12*	Internal Forward (5'-3')	CTGGAGGAAGAGTCAGCCTTG		

* Downstream exon 12
